# Determinants of mental health among youths and adolescents in the digital era: Roles of cyber and traditional bullying, violence, loneliness, and environment factors

**DOI:** 10.3389/fpubh.2022.971487

**Published:** 2022-10-25

**Authors:** Tam Chi Phan, Brandon Chau, Ha Ngoc Do, Thao Bich Thi Vu, Khanh Long Vu, Hiep Duy Nguyen, Dung Tuan Nguyen, Hoang Minh Do, Nga Thu Thi Nguyen, Ly Bac Thi La, Vu Anh Trong Dam, Hien Thu Nguyen, Long Hoang Nguyen, Anh Linh Do, Thuc Minh Thi Vu, Minh Ngoc Vu Le, Giang Thu Vu, Linh Khanh Le, Carl A. Latkin, Cyrus S. H. Ho, Roger C. M. Ho

**Affiliations:** ^1^School of Pharmacy, University of Southern California, Los Angeles, CA, United States; ^2^Keck School of Medicine, University of Southern California, Los Angeles, CA, United States; ^3^Youth Research Institute, Ho Chi Minh Communist Youth Union, Vietnam Youth Academy, Hanoi, Vietnam; ^4^Department of Research on Youth's Organisations and Youth Campaign, Youth Research Institute, Ho Chi Minh Communist Youth Union, Hanoi, Vietnam; ^5^Department of Research on Children's Issues, Youth Research Institute, Ho Chi Minh Communist Youth Union, Hanoi, Vietnam; ^6^Department of Research on Youth and Legal Issues, Youth Research Institute, Ho Chi Minh Communist Youth Union, Hanoi, Vietnam; ^7^Department of Research on Youth Culture and Lifestyle, Youth Research Institute, Ho Chi Minh Communist Youth Union, Hanoi, Vietnam; ^8^Faculty of Social Sciences and Humanities, Hanoi Metropolitan University, Hanoi, Vietnam; ^9^Faculty of Preschool Education, Hanoi National University of Education, Hanoi, Vietnam; ^10^Institute for Global Health Innovations, Duy Tan University, Da Nang, Vietnam; ^11^Faculty of Nursing, Duy Tan University, Da Nang, Vietnam; ^12^Department of Public Health Sciences, Karolinska Institute, Stockholm, Sweden; ^13^Institute of Health Economics and Technology, Hanoi, Vietnam; ^14^Center of Excellence in Evidence-Based Medicine, Nguyen Tat Thanh University, Ho Chi Minh City, Vietnam; ^15^Business Management Department, Troy University, Troy, AL, United States; ^16^Bloomberg School of Public Health, Johns Hopkins University, Baltimore, MD, United States; ^17^Department of Psychological Medicine, Yong Loo Lin School of Medicine, National University of Singapore, Singapore, Singapore; ^18^Institute for Health Innovation and Technology (iHealthtech), National University of Singapore, Singapore, Singapore

**Keywords:** mental health, adolescents, traditional bullying, violence, loneliness, youths, environmental factors

## Abstract

**Introduction:**

Although the pathogenesis of depressive disorders is not fully elucidated, untreated adolescent depression can lead to serious sequelae such as impaired academic performance and social functioning, substance use disorders, poor self-esteem, and increased risk for suicidal ideation and attempts. Literature on adolescent mental health in Vietnam is limited, despite increased international awareness of this critical issue. This study aimed to investigate the prevalence and associations of depressive symptoms in Vietnamese adolescents.

**Methods:**

A cross-sectional, self-administered survey was conducted in five provinces of Vietnam among adolescents aged 14 to 24 years. In addition to collecting participants' demographics, a structured questionnaire was developed to examine depressive symptoms, suicidal ideation, loneliness, and cyberbullying victimization. Depressive symptoms and loneliness were assessed *via* the PHQ-9 and UCLA Loneliness Scale, respectively. Two-tailed Chi-squared, Mann-Whitney, and Kruskal-Wallis tests were performed to examine associations between variables. Multivariate Logistic regression models were conducted to examine the associations between prior-defined variables and positive depressive symptoms.

**Results:**

Among 1,600 respondents, 31.8% of participants reported having mild-moderate or severe depressive symptoms. Participants within the moderate-severe depressive symptom group had significantly lower community cohesion scores than those of participants in normal and mild depressive symptom groups (*p* < 0.05). Youths living alone were more likely to have moderate-severe depressive symptoms (OR 2.16; 95% CI: 1.09–4.25). Cyberbullying had significant associations with depressive severity (OR 1.93; 95% CI 1.38–2.70).

**Conclusion:**

The findings of this study characterize various risk and protective factors for depression in Vietnamese youths and adolescents. The results highlight the importance of raising awareness and increasing access to educational resources for depression and other mental health illnesses. With the rising prevalence of depression, parents, teachers, and community leaders play a vital role in addressing mental health problems in adolescents.

## Introduction

Major depression and mood disorders are complex mental health problems associated with changes in mood, thinking, motivation, and behavior. The etiology of mood disorders is multifactorial, with an increasing prevalence of serious psychological distress, depression, suicidal ideation, and behaviors among youths and adolescents from 2005 to 2017 (1). Left untreated, depression in adolescents and youths may lead to impaired academic performance and social functioning, substance use disorders, poor self-esteem, and increased risk for suicidal ideation and attempts (2–5). Major depression is a strong risk factor for suicide, given that 90% of adolescents and youths who died from suicide have been diagnosed with a psychiatric disorder (6). Previous data has shown that up to 50% of depressed adolescents will attempt suicide with an 8% completion rate over a decade (6). Major depression in youth and adolescent populations is often a chronic condition, with recurrence rates reaching 45% within 6 years of the index episode (7). According to the World Health Organization (WHO), suicide is the second leading cause of self-inflicted death in 15–29-year-olds and the third leading cause among those 15–44 years old (8).

Adolescence is an ideal time to develop a self-identity and establish interpersonal relationships and a sense of belonging for an individual (9, 10). Unfortunately, depression in children is often overlooked because the presentation of symptoms can differ from that of adults; indeed, only 50% of adolescents are properly diagnosed before adulthood (5). Furthermore, adolescent depression can vary among age groups, with different signs and symptoms (11). Additionally, adolescent patients often do not disclose the full extent of psychiatric symptoms for fear of the stigma attached to more severe psychiatric conditions (12). Given the complexity of major depression pathogenesis and the impact of increasing technological access, previous studies have characterized factors such as loneliness, violence, living conditions, and bullying and their roles in depression.

In addition to biomedical risk factors, screening for adolescent depression should also consider environmental and psychosocial risk factors. Previous studies have demonstrated that bullying and other adverse childhood experiences are risk factors for depression and suicidal ideation (13–17). Furthermore, feelings of loneliness have been cited as an additional risk factor for depression (18, 19). Factors such as low socioeconomic status and living conditions also influence one's mental health, with prior studies correlating loud noises, pollution, and living in rural areas with depressive symptoms (20–22). In addition, with the advent of technology, the role of cyberbullying as another platform to experience these adverse childhood experiences has been assessed (23). As technology continues to become more prevalent in youths and adolescents' lives, Sampasa-Kanyinga et al. have demonstrated that depression mediates bullying victimization, both traditional and virtual, as well as suicide attempts, which extends from previous studies to also discuss cyberbullying (24). Potentially, the interplay between traditional physical bullying and cyberbullying may pose a synergistic assault on the developing child. To date, there is a strong association between bullying victimization and a greater likelihood of having depressive symptoms, suicidal ideation, and suicide attempts (25). However, previous literature characterizing these relationships in Vietnamese youths and adolescents remains limited with opportunities for improvement. A systematic review conducted in 2019 indicated there are no recent studies looking at the impact of bullying (traditional and cyber) on Vietnamese children, while a cross-sectional analysis performed by Nguyen et al. only included a sample from one center in Can Tho City (26).

With increasing awareness of mental health on a global scale, developing countries such as Vietnam are pursuing various endeavors to strategically provide culture-specific interventions (27, 28). Vietnam is a rising economy with correlating socio-economic reforms, demonstrated by increased internal migration to rural areas, and changes in family structure and dynamics (29, 30). Several studies have shown that anxiety, depression, and suicidal ideation are common issues among adolescents in several provinces of Vietnam (31–33). However, there still exists a knowledge gap in characterizing depression in youths and adolescents in Vietnam. In our study, we used multistage analysis and put contextual factors such as environmental factors and psychosocial risk factors to analyze their influence on depression. Hence, our study provides a comprehensive picture of the mental health of adolescents in relation to the roles of cyber and traditional bullying, violence, loneliness, and environmental factors. This study examines the prevalence of depressive symptoms with respect to cyberbullying victimization, the experience of loneliness, violence, self-perceived health status, substance use, and community cohesion across five different regions in Vietnam.

## Methods

### Study design, sampling method, and data collection

The cross-sectional study was used in five provinces/cities of Vietnam: Ha Noi, Ho Chi Minh, Tuyen Quang, Quang Tri, and Dak Lak from January to December 2019. We recruited participants who (1) were youths or adolescents (14–24 years old); (2) were current Internet users, and (3) agreed to participate and give their informed consent. If their age was < 18 years (about 21.1%), agreements from their parents or guardians were sought (*via* Ho Chi Minh Communist Youth Union). Stratified random sampling combined with cluster sampling was used to recruit participants for the study. A total of 1,600 participants (320 participants/province) were invited.

Participants were given a brief overview of the study before being asked to sign written informed consent. Participants were given a brief overview of the study before being asked to sign written informed consent. We did not collect any identifiable information about the participant for facilitating their enrollment and preventing any social desirability bias.

### Instruments

The data was collected by using a structured questionnaire. The questionnaire included information about the socio-economic status (gender (Male/Female), age, marital status (Single/Having Partner/Being Married), living arrangement (Family/Friend/Alone), living location (Urban/Rural/Mountainous), social support and community cohesion, health characteristics (psychological distress and self-reported health status), substance use behaviors, cyberbullying victimization, loneliness, and violence.

#### Depressive symptoms

We used the Patient Health Questionnaire (PHQ-9) to evaluate the depressive symptoms of the participants. This tool was developed based on the standard of the Diagnostic and Statistical Manual of Mental Disorders, 5th Edition (DSM-V), and has been widely used in Vietnam (34–36). This instrument has nine items mentioning the frequency of different depressive symptoms that the participants might have suffered in the last 2 weeks. There are four options to answer, corresponding to scores from 0 (Not at all) to three (Nearly every day). The score ranges from 0 to 27 (37). Following cut-off thresholds were used: Normal/Minimal (0–4), Mild (5–9), Moderate (10–14), Moderately Severe (15–19), and Severe (20–27) (37). Patients were categorized into a “positive depressive symptom” group if they had 10 points or more (38–40).

#### Cyberbullying victimization

We developed a new questionnaire based on literature view and expert consultancy. There were 13 items in this questionnaire regarding different aspects of cyberbullying victimization. Each item had five levels of response from one “Never” to five “Always.” There were three domains extracted after the factor analysis including Dissing/Flaming (four items, score ranges from one to five), Harassment (six items, score ranges from one to five), and Exclusion/Framing (three items, score ranges from one to five). The Cronbach's alpha of Dissing/Flaming, Harassment, and Exclusion/Framing was 0.75, 0.84, and 0.82, respectively.

#### Violence

In this study, we developed an instrument to measure violence among youths. We reviewed the literature and focused on physical and verbal violence, as well as self-harm as common types of violence in youths and adolescents. A total of 15 items were collected to construct the instrument. Participants were asked to report the frequency of following actions in recent times, with four options from one (Never), two (Rarely), three (Sometimes), and four (Often):

1) Make noise, shout angrily
2) Scolding others with words that insult themselves (to a mild extent)
3) Profanity, profanity, malicious curse, intimidation of others or yourself (moderately)
4) Make threats of violence obvious, such as would harm others
5) Graffiti, throwing objects but not damaging
6) Smash the door, throw loose clothes, make a mess
7) Break objects, kick walls, smash windows
8) Scratching your skin, hitting yourself, and pulling on your hair (without or only causing minor injuries)
9) Smashing heads, punching objects on objects, slamming things on the floor or objects (painful but not dangerous injuries)
10) Cause cuts, bruises, or minor burns to yourself
11) Self-harming: causes deep cuts, bleeds, causes serious wounds, fractures, etc.
12) Do things that only threaten and nudge at other friends
13) Attacking, kicking, pushing, pulling other people's hair but not causing them injury
14) Attacking others, causing mild, moderate injuries (bruises, sprains,)
15) Attacking others, causing serious physical injuries (broken bones, causing serious injuries).

Based on the factor loading, there were three domains including Self-harm (seven items), Physical violence (four items), and Verbal violence (four items). The rating for each domain ranges from one to four. These scores were higher than the level of violence was higher. The Cronbach's alpha of Self-harm, Physical violence, and Verbal violence was 0.89, 0.90, and 0.79, respectively.

#### Loneliness

A UCLA Loneliness Scale was used to measure one's subjective feelings of loneliness as well as feelings of social isolation. Originally, this instrument had 20 items. However, after consulting with experts in the field of youths and adolescents, we adapted this instrument by removing the item “How often do you feel that you are no longer close to anyone?” which was suggested to be very similar to another item “How often do you feel close to people?.” Then, a new instrument was constructed including 19 items. Participants rated each item on a scale from one (Never) to four (Often). There were three domains including Loneliness and Isolation (10 items, score ranges from one to four, a higher score means a higher level of loneliness and isolation), Sociability (five items, score ranges from one to four, a higher score means a higher level of unsociability), and Companionship (four items, score ranges from one to four, a higher score means a lower level of companionship). The Cronbach's alpha of Loneliness and Isolation, Sociability, Companionship was 0.87, 0.82, and 0.73, respectively.

#### Health status

We measured the self-rate health by employing a Visual analog scale (VAS), with a range score from 0 (The worst health state that you can imagine) to 100 (The best health state that you can imagine).

#### Substance use behaviors

Participants were asked to report whether they used alcohol or not; and whether they smoked tobacco in the last 30 days or not.

#### Community cohesion

Participants were asked to show their attitude about the community cohesion around their current living area [including neighborhood cohesion (41), neighborhood disorder (42), and social disorder (42)]. The issues that the participants were asked included: About the people around (1) willing to help each other, (2) willing to help neighbors, (3) living in harmony, (4) being trusted and reliable, (5) sharing the same value and life concept, and about the area where you live (1) a tremendous amount of trash, (2) society's vices, (3) many fights and quarrels.

### Statistical analysis

STATA software 15.0 was used for all analyses. The alpha level for statistical significance was set at 0.05. Two-tailed Chi-squared, Mann-Whitney, and Kruskal-Wallis tests were performed to examine differences in sociodemographic characteristics, community characteristics, cyberbullying, violence, and loneliness regarding different depressive severity and suicidal ideation. Multivariate Logistic regression models were conducted to examine the associations between prior-defined variables and Depressive severity. We selected the models by a backward stepwise selection strategy. Variables were excluded when those *p*-values of the log-likelihood ratio test were more than 0.2.

### Reliability

The internal consistency reliability was checked by calculating Cronbach's alpha. The alpha value of 0.7 or above was considered an acceptable (43). Additionally, we also assessed domain-domain correlation, item-item correlation, item-total correlation, and Cronbach's alpha of the domain if the item was deleted.

## Results

Table 1 shows the characteristics of 1,600 respondents. In total, there were 31.8% had mild-moderate or severe depressive symptoms. Differences in depressive severity were found regarding age groups, sex, education, living arrangement, province, and location (*p* < 0.05).

**Table 1:** Sociodemographic and community characteristics.

**Characteristics**		**Depressive severity**						***p*-value**
	**Total**	**Normal**		**Mild**		**Moderate to Severe**		
	***n***	***n***	**%**	***n***	**%**	***n***	**%**	
**Total**	1,600	1,091	68.2	370	23.1	139	8.7	
**Individual characteristics**								
**Age group**								
14–17 years old	385	283	25.9	80	21.6	22	15.8	**< 0.01**
18–20 years old	914	643	58.9	198	53.5	73	52.5	
21–24 years old	301	165	15.1	92	24.9	44	31.7	
**Sex**								
Male	626	475	43.5	109	29.5	42	30.2	**< 0.01**
Female	974	616	56.5	261	70.5	97	69.8	
**Education**								
High school	550	429	39.3	90	24.3	31	22.3	**< 0.01**
Student	1,050	662	60.7	280	75.7	108	77.7	
**Living arrangement**								
Family	1,269	871	79.8	290	78.4	108	77.7	**< 0.01**
Friends	205	151	13.8	47	12.7	7	5.0	
Alone	126	69	6.3	33	8.9	24	17.3	
Substance use*	288	193	17.7	71	19.2	24	17.3	0.79
**Community characteristics**								
**Province**								
Tuyen Quang	320	154	14.1	120	32.4	46	33.1	**< 0.01**
Ha Noi	320	228	20.9	69	18.7	23	16.6	
Quang Tri	320	194	17.8	83	22.4	43	30.9	
Dak Lak	320	249	22.8	55	14.9	16	11.5	
Ho Chi Minh city	320	266	24.4	43	11.6	11	7.9	
**Location**								
Urban	1,039	725	66.5	220	59.5	94	67.6	**0.04**
Rural mountainous	561	366	33.6	150	40.5	45	32.4	

*Alcohol use or tobacco smoking. The bold values indicate the difference is statistically significant.

Regarding neighborhood characteristics, Table 2 shows that respondents with severe depressive symptoms were less likely to live in a community where “people around can be trusted and reliable” (20.1%) but were more likely to live in a place having “a tremendous amount of trash,” and “society's vices” compared to other groups (*p* < 0.05). The community cohesion score in severe depressive symptom groups was significantly lower than that of other groups (*p* < 0.05).

**Table 2:** Neighborhood characteristics of respondents.

**Characteristics**		**Depressive severity**						***p*-value**
	**Total**	**Normal**		**Mild**		**Moderate to severe**		
	***n***	***n***	**%**	***n***	**%**	***n***	**%**	
People in this area are willing to help each other	893	579	53.1	222	60.0	92	66.2	**< 0.01**
People around living place are willing to help neighbors	899	619	56.7	199	53.8	81	58.3	0.54
People live in harmony	936	650	59.6	208	56.2	78	56.1	0.44
People around can be trusted and reliable	635	438	40.2	169	45.7	28	20.1	**< 0.01**
People share the same value, life concept	477	341	31.3	101	27.3	35	25.2	0.16
This area has a tremendous amount of trash	185	100	9.2	32	8.7	53	38.1	**< 0.01**
This area has society's vices	157	61	5.6	32	8.7	64	46.0	**< 0.01**
This area has many fights and quarrels	53	30	2.8	15	4.1	8	5.8	0.12
		$\bar{X}$	**SD**	$\bar{X}$	**SD**	$\bar{X}$	**SD**	***p*** **-value**
Community cohesion		5.2	1.6	5.2	1.7	4.4	1.4	**< 0.01**

The bold values indicate the difference is statistically significant.

Table 3 presents that normal youths have lower scores in all cyberbullying, loneliness, and violence domains compared to other groups, except companionship (*p* < 0.05).

**Table 3:** Cyberbullying, loneliness, and violence among respondents.

**Characteristics**	**Depressive severity**						***p*-value**
	**Normal**		**Mild**		**Moderate to severe**		
	** $\bar{x}$ **	**SD**	** $\bar{x}$ **	**SD**	** $\bar{x}$ **	**SD**	
**Cyberbullying**							
Dissing/flaming	1.4	0.5	1.6	0.7	1.9	0.8	**< 0.01**
Harassment	1.4	0.5	1.7	0.8	1.8	0.9	**< 0.01**
Exclusion/fraping	1.5	0.8	1.7	0.9	1.9	1.0	**< 0.01**
**Loneliness**							
Sociability	1.8	0.7	2.1	0.8	1.9	0.6	**< 0.01**
Companionship	2.3	0.8	2.6	0.7	2.2	0.7	**< 0.01**
Loneliness and isolation	1.9	0.6	2.2	0.6	2.7	0.5	**< 0.01**
**Violence**							
Verbal	1.3	0.4	1.6	0.6	2.0	0.6	**< 0.01**
Physical	1.1	0.3	1.4	0.7	1.3	0.6	**< 0.01**
Self-harm	1.1	0.3	1.4	0.6	1.4	0.5	**< 0.01**

The bold values indicate the difference is statistically significant.

Factors associated with depressive symptoms are presented in Table 4. Youths living alone were more likely to have depressive symptoms. Meanwhile, those with high self-reported health status and high community cohesion scores had a lower likelihood of having depressive symptoms. A higher level of exclusion/framing, loneliness and isolation, and verbal violence were positively associated with depressive symptoms, while the higher level of physical violence was negatively related to depressive symptoms.

**Table 4:** Associated factors with depressive symptoms.

**Characteristics**	**Depressive symptoms**	
	**Adjusted OR**	**95%CI**
**Sociodemographic, behavioral and health characteristics**		
Age (per year)	1.17*	0.97–1.40
Gender (Female vs. male-ref)	0.90	0.54–1.52
Education (University student vs. high school-ref)	0.81	0.37–1.77
**Living arrangement (Family-ref)**		
Friends	0.55	0.22–1.38
Alone	2.16**	1.09–4.25
Substance use^a^ (Yes vs. No-ref)	0.69	0.37–1.27
Self-reported health status (0–100)	0.97***	0.95–0.98
Location (Rural mountainous vs. Urban-ref)	0.73	0.42–1.28
**Province (Tuyen Quang-ref)**		
Ha Noi	0.59	0.23–1.52
Quang Tri	1.46	0.67–3.20
Dak Lak	0.32**	0.13–0.79
Ho Chi Minh city	0.16***	0.06–0.45
Community cohesion score (per score)	0.72***	0.61–0.85
Cyberbullying victimization, loneliness, and violence		
**Cyberbullying victimization**		
Dissing/flaming (per score)	1.61*	0.92–2.81
Harassment (per score)	0.73	0.40–1.33
Exclusion/fraping (per score)	1.93***	1.38–2.70
**Loneliness**		
Sociability (per score)	0.69	0.43–1.10
Companionship (per score)	0.86	0.57–1.29
Loneliness and Isolation (per score)	5.22***	3.30–8.26
**Violence**		
Verbal (per score)	3.48***	2.20–5.50
Physical (per score)	0.55**	0.33–0.94
Self-harm (per score)	0.64	0.34–1.20

*** p < 0.01, ** p < 0.05, * p < 0.1.

^*a*^Acohol use or tobacco smoking.

## Discussion

This study investigated the associations of depressive symptoms concerning social demographics, loneliness, community cohesion, cyberbullying, and violence among Vietnamese adolescents. With increasing awareness of mental health and the potential long-term impact for adolescents, it is imperative to characterize protective and risk factors for depression.

In our nationwide survey, we categorized depressive symptoms based on PHQ-9 scores and their classification of depression. Overall, 8.7% (*n* = 139) of our study participants reported moderate to severe depressive symptoms across Ha Noi, Quang Tri, Dak Lak, and Ho Chi Minh City. In contrast, a previous study conducted by Nguyen and colleagues showed that 41.1% of responders with depressive symptoms (31). However, the study surveyed secondary school students (age 15–19) in Can Tho City, which may only demonstrate a limited sample of a population. In the present study, living in Dak Lak (OR 0.32, 95% CI 0.13–0.79) and Ho Chi Minh City (OR 0.16, 95% CI 0.06–0.45) was associated with significantly lower odds of having depressive symptoms while living in Dak Lak (OR 0.24, 95% CI 0.12–0.47) was associated with lower odds of having suicidal ideation. In comparison, a study in 2007 by Banh showed a lower prevalence of suicidal ideation across these Ho Chi Minh and Hanoi, 6.3 and 10.6%, respectively (44). Local, regional, and global variations in mental health statuses in youth and adolescents remain complex and can be attributed to several factors including cultural stressors, or environmental factors such as traumatic events and poverty (23). Socio-economic development status in industrialized cities such as Ho Chi Minh may explain the lower prevalence of depression.

Cyberbullying had significant associations with depressive severity, and higher levels of exclusion/framing were positively associated with depressive symptoms. This is largely consistent with past studies, which indicate that cyberbullying victimization is positively correlated with depression (45). However, recent rates of cyberbullying in Vietnam have become alarmingly high (up to 24%), but little has been done to address this issue at the level of policy and public health (46). Although access to technology can be largely attributed to rising rates of cyberbullying, digital literacy in Vietnamese society plays an extremely important role. Defined as the ability to effectively navigate and evaluate information on media and digital platforms, digital literacy is an important skill for all those using online technologies, as it promotes the ethical and responsible use of media. Learning to safely access the internet can have protective factors against cyberbullying that will prove useful in the face of the inevitably expanding access to technology during adolescence, a particularly vulnerable time in development (47, 48). Caretakers and educators need to be more informed about adolescent access to technology and their awareness of the risks associated with media consumption.

Additionally, interpersonal relationships can be immensely influential on the mental health of youths and adolescents. Having trusted social connections was a major protective factor in the present study. When comparing the aggregate community cohesion scores among normal, mild, and severe depressive severity, those who had severe depressive symptoms also had lower community cohesion scores (4.4 +/– 1.4; *p* < 0.01). Further, previous research has linked exposure to violence and poor school or familial relationships to an increased risk for depression.

Moreover, experiencing bullying and living around violence can create a stressful environment for students, which fosters strong feelings of insecurity at school or at home. Consequently, this could lead to truancy from school or isolation from friends, teachers, or family members. In detrimental interpersonal relationships, such as traditional domestic violence in Vietnamese households and communities, children's coping strategies can largely be categorized as emotional-focused coping (managing/reducing stress) or problem-focused coping (changing the problematic situation) (49). Social isolation and increased internet usage are examples of emotional-focused coping strategies to mitigate negative feelings of sadness, anxiousness, or loneliness (50, 51). Increased internet usage can socially distance peers and fragment social cohesion, which has been shown to be a major protective factor against moderate to severe depressive symptoms in our study. Additionally, increased time on the internet can lead to more numerous exposures to cyberbullying, which can provide additional risk factors for moderate to severe depressive symptoms. Although traditionally bullying and cyberbullying can often occur in conjunction with one another, cyberbullying may leave digital footprints that can be reported to family members, teachers, or other community members. As a result, interventions to address the duration of internet use, educate users on social media etiquette, how to report incidents of cyberbullying, and spread awareness among community members are all effective interventions.

Potentially, interventions to raise community cohesion scores may address social fragmentation, which has been shown to be associated with depression, suicidality, and mental health (48–50). On the other hand, participants who had major depressive severity also reported living in areas where there were fights, excessive trash, and society's vices, which have also been reported in Vietnam to have greater associations with individuals living with depression (3). Our results confirmed that social capital and living conditions have an immense effect on depressive severity, which underscores the need for community policies and interventions. These highly modifiable factors have great potential as a cost-effective avenue for improving mental health amongst youth and should receive greater attention from policymakers.

Beyond digital literacy, efforts to improve mental health on local, regional, and national levels can provide a cost-effective and sustaining intervention to detect and prevent the possible development of mental health disorders. Mental health literacy is described as an individual's knowledge about mental illnesses that can help in recognizing, managing, and preventing these issues. On an individual level, personal and cultural biases can skew the perception of psychiatric symptoms, which in turn may prevent proper recognition of the clinical features of common mental health disorders. In a survey of undergraduate students in Hanoi by Thai and Nguyen in 2018, 81.1% of respondents were unable to properly recognize signs of depression (48). This major deficit is a unique opportunity for educators and policy-makers to improve child and adolescent mental health across the nation, as improvement of mental health relies largely on detection and recognition of disorders while providing low-cost resources and educating families. Extending beyond the physical environment, the present study shows the internet is a novel platform, where students may encounter these intentionally aggressive behaviors. However, prior research in other countries has shown that incorporating positive technology or mobile apps can be effective mobile-based interventions with high levels of receptiveness and satisfaction (52). Given that adolescent mental health is influenced by a variety of factors, educational resources should also be made available to parents, teachers, and community leaders to increase awareness of the issue and encourage adults to take an active role in intervention upon identification of signs of depression or bullying.

This study is one of the initial studies to characterize associations between depressive symptoms and suicidal ideation in Vietnamese adolescents, but the research has limitations. First, the study is a cross-sectional analysis; as a result, a causal relationship of one factor with depression or suicidal ideation is not established, and related factors may not be clearly explained. Further, since this study pertains to adolescents, longitudinal data for future studies can fill in gaps missing from a cross-sectional analysis as the youth develop. Although there are a variety of quantitative factors presented, qualitative responses and subsequent thematic analysis of qualitative data will make policy development more pragmatic and culturally sensitive. Given that depression is inherently a sensitive topic, conducting anonymous data collection can lead to inaccurate clerical errors in terms of data entry. To remedy these potential errors, researchers worked closely with the school to collect informed consent. Future studies can assess both qualitative perceptions from adolescents and potentially follow participants over a period of time to collect longitudinal data.

## Conclusion

In the present study, the prevalence of depression among youths and adolescents is relatively high in Vietnam. Investigating further into potential associated factors indicate positive correlations with participants' demographic, geographical living location, community characteristic, interpersonal relationships, internet usage, and experience of bullying, both traditional and online. Efforts to improve health and digital literacy in Vietnamese youth, regular mental health screenings, and social support are important factors that should receive more attention from community leaders, educators, and policy-makers in developing nationwide interventions. These findings suggest that policy and guidelines to address mental health problems in Vietnamese youths and adolescents should be cognizant of specific needs and limitations of the various diverse populations in different regions of Vietnam. Lastly, these interventions should be multimodal, evidence-based, and culturally inclusive of the intended beneficiary.

## Data availability statement

The original contributions presented in the study are included in the article/supplementary material, further inquiries can be directed to the corresponding author.

## Ethics statement

This study was approved by the Institutional Review Board of the Youth Research Institute and performed according to the Helsinki Declaration Guideline. The patients/participants provided their written informed consent to participate in this study.

## Author contributions

Conceptualization: TP, BC, VD, LBTL, TMTV, MV, GV, LKL, and CH. Data curation: TP, BC, HND, TBTV, DN, HMD, and NN. Formal analysis: KV, HDN, DN, HMD, NN, LBTL, TMTV, and AD. Investigation: KV, HDN, DN, LN, AD, CL, and RH. Methodology: HND, TBTV, LN, HTN, HMD, NN, and AD. Supervision: HTN, VD, HND, TBTV, KV, HDN, MV, and LKL. Writing—original draft: HTN, GV, LKL, CL, RH, and CH. Writing—review and editing: TP, BC, HTN, VD, LBTL, TMTV, MV, GV, CL, RH, and CH. All authors contributed to the article and approved the submitted version.

## Funding

The article process charge of this paper is supported by NUS Department of Psychological Medicine (R-177-000-100-001/ R-177-000-003-001/R177000702733) and NUS iHeathtech Other Operating Expenses (R-722-000-004-731).

## Conflict of interest

The authors declare that the research was conducted in the absence of any commercial or financial relationships that could be construed as a potential conflict of interest.

## Publisher's note

All claims expressed in this article are solely those of the authors and do not necessarily represent those of their affiliated organizations, or those of the publisher, the editors and the reviewers. Any product that may be evaluated in this article, or claim that may be made by its manufacturer, is not guaranteed or endorsed by the publisher.
